# Effects of intraocular treatments for Epstein-Barr virus (EBV) retinitis: A case report

**DOI:** 10.1097/MD.0000000000028101

**Published:** 2021-12-03

**Authors:** Yasuaki Mushiga, Tatsunori Komoto, Norihiro Nagai, Yoko Ozawa

**Affiliations:** a Department of Ophthalmology, St. Luke's International Hospital, 9-1 Akashi-cho, Chuo-ku, Tokyo, Japan; b Laboratory of Retinal Cell Biology, St. Luke's International University, 9-1 Akashi-cho, Chuo-ku, Tokyo, Japan; c Department of Ophthalmology, Keio University School of Medicine, 35 Shinanomachi, Shinjuku-ku, Tokyo, Japan.

**Keywords:** epstein–barr virus, foscarnet, inflammation, methotrexate, optic nerve, retina

## Abstract

**Rationale:**

Intraocular infection of Epstein–Barr virus (EBV) may cause severe visual loss. However, it is relatively rare, and there is no consensus on its treatment.

**Patient concerns:**

A 44-year-old woman complained of a right-eye floater and exhibited a unilateral exudative change along the retinal veins at the Department of Ophthalmology, St. Luke's International Hospital.

**Diagnosis:**

EBV retinitis was diagnosed based on EBV-positive (9.09 × 10^3^ copies/μl) and cytomegalovirus-negative results in the aqueous humor.

**Interventions:**

Oral prescription of valaciclovir hydrochloride, and an intravitreal injection of foscarnet sodium hydrate was administered. However, the retinal infiltration progressed, and vitreous opacity with cellular infiltration appeared. Intravitreal methotrexate (MTX) injection effectively suppressed retinal and vitreous infiltration. However, she developed optic-nerve papillitis, and central retinal vein occlusion related to the severe swelling of the optic-nerve, and began steroid pulse therapy. Considering the increase in intraocular EBV levels to 6.4 × 10^4^ copies/ml, we restarted intravitreal foscarnet injections replacing MTX. This in turn rapidly reduced the EBV levels to 3.27 × 10^4^ copies/ml, followed by papillitis alleviation.

**Outcomes:**

The intraocular MTX administration reduced the inflammatory vitreous and retinal infiltration, but not the EBV load, while foscarnet reduced the EBV load and papillitis, but not vitreous infiltration.

**Lessons:**

The retinal infiltration may have involved EBV infection to the retinal neurons but also EBV-free reactive inflammatory cells. EBV infection to the neurons may have been, at least partially, treated by intravitreal foscarnet treatment, and the reactive inflammatory cells by intravitreal MTX. Further observations are warranted to reach a consensus on treating intraocular EBV infection.

## 1 Introduction

Intraocular infection caused by the Epstein–Barr virus (EBV) may result in severe visual loss. However, it is relatively rare, and there is no consensus on its treatment. This may be partially because of the unclear effects and roles of drugs tried for the treatment. Herein, we presented a case of EBV retinitis and optic-nerve papillitis, and the intraocular viral levels over time. This report will elucidate the role of each drug for the treatment of EB viral infection.

The EBV, formally called the Human gammaherpesvirus 4, is a double-stranded DNA virus of the Herpesviridae family. Over 90% of adults have been infected with EBV worldwide, and carry the virus as a lifelong persistent infection.^[2]^ EBV principally infects B cells of the immune system and epithelial cells. While it may cause chronic intraocular inflammation, such as multifocal choroiditis,^[9]^ it can also cause acute retinal necrosis that severely affects the retinal neurons^[6]^ and optic neuritis,^[5]^ both of which may cause blindness.

## 2 Case presentation

**Figure 1:**
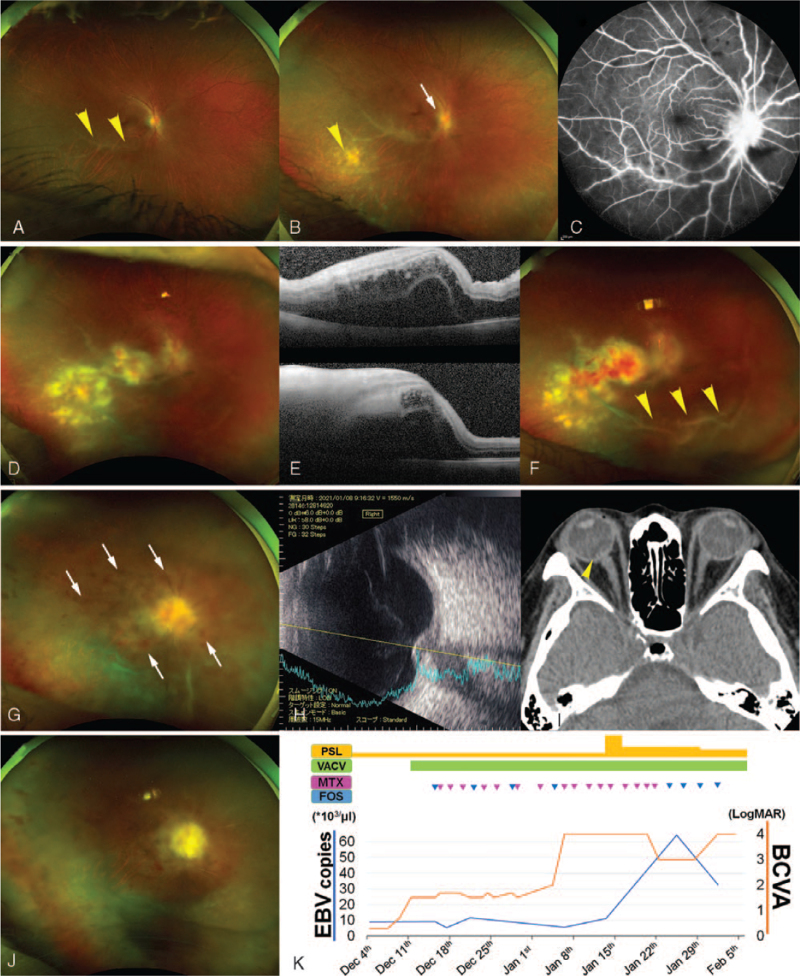
Clinical course of the patient with Epstein–Barr virus intraocular infection. A unilateral exudative change along the vessel appears at the temporal retina of the 44-year-old patient on October 30, 2020 (A, arrowheads). A snowball-like vitreous opacity, recent retinal spot (B, arrowhead), and peripapillary exudative changes (B, arrow) have developed by November 26, 2020 (B), detected on fluorescein angiography (C). By December 11, 2020, the retinal lesions, such as retinal infiltration and hemorrhage, have progressed to involve the macula (D), with severe edema and exudative detachment observed on optical coherence tomography (E, upper, horizontal image; lower, vertical image). Retinal hemorrhage and vitreous opacity have been observed on December 14, 2020 (F, arrowheads). On January 6, 2021, she has developed central retinal vein occlusion (G, hemorrhage is pointed by arrows) related to the swelling of the optic-nerve papillae recorded, using echography (H) and computed tomography (I, arrowhead). However, the vitreous infiltration is suppressed (G). The activity of the optic-nerve lesion and the retinitis and vitreous infiltration are suppressed on January 28, 2021 (J). (K) depicts the EBV copy numbers in the aqueous humor and best-corrected visual acuity over time, in relation to the administered drugs. Each triangle denotes the intravitreal injection (MTX, pink; foscarnet, blue). EBV = Epstein–Barr virus, FOS = foscarnet, MTX = methotrexate, VACV = valaciclovir hydrochloride.

A 44-year-old woman was being inspected for chloroquine and 6 mg oral prednisolone toxicities used to treat systemic lupus erythematosus, at the Department of Ophthalmology, St. Luke's International Hospital. On October 30, 2020, she complained of a right-eye floater, and exhibited a unilateral exudative change along the inferior temporal retinal veins (Fig. 1A), without anterior-segment inflammation. Her best-corrected visual acuity (BCVA) was -0.079 (LogMAR) and normal. She had a history of renal dysfunction and bilateral femoral-head necrosis because of systemic lupus erythematosus and long-term steroid use.

Her BCVA had declined to 0.301 by November 26, 2020. She exhibited keratic precipitates, snowball-like vitreous opacity, and recently appeared retinal and peripapillary exudative spots (Fig. 1B). Fluorescein angiography displayed intensive vascular leakage in the retina and at the optic-nerve papillae (Fig. 1 C). She was suspected to have cytomegalovirus retinitis, and was prescribed half-dose oral valganciclovir hydrochloride daily. She was administered 20 mg of a sub–tenon injection of a steroid, triamcinolone acetonide, considering her renal dysfunction and refusal to get hospitalized. By December 11, 2020, her BCVA declined to 0.699. This is because the retinal infiltration and retinal hemorrhage expanded to cause macular edema (Fig. 1D, E). EBV retinitis was diagnosed based on EBV-positive (9.09 × 10^3^ copies/μl) and cytomegalovirus-negative results in the aqueous humor. She demonstrated positive serum EBV results. Herpes-simplex virus, varicella-zoster virus, and toxoplasma gondii were negative in the aqueous humor. We changed the oral prescription to half-dose valaciclovir hydrochloride, which can be sensitive to EBV,^[8,10]^ and added an intravitreal injection of 1.2 mg foscarnet sodium hydrate once a week, referring to previous reports.^[1,4]^ By December 14, 2020, the retinal infiltration had progressed, and vitreous opacity with cellular infiltration had appeared and progressively increased (Fig. 1F). Referring to previous reports on intraocular EBV infection,^[1,4]^ we prescribed 0.4 mg intravitreal methotrexate (MTX) injection, twice a week, which effectively suppressed retinal and vitreous infiltration. However, by January 6, 2021, her BCVA had declined to light perception because of optic-nerve papillitis. We replaced the foscarnet injection with MTX to be administered thrice-weekly. However, on January 11, 2021, she developed central retinal vein occlusion and resulting retinal hemorrhage (Fig. 1G), which is related to severe swelling of the optic-nerve papilla where the vein passes through (Fig. 1H, I). She underwent steroid pulse therapy for treating optic-nerve inflammation on January 14, 2021. Considering the increase in intraocular EBV levels to 6.4 × 10^4^ copies/ml on January 25, 2021, we restarted intravitreal foscarnet injections replacing MTX, twice-weekly. This in turn rapidly reduced the EBV levels to 3.27 × 10^4^ copies/ml, followed by papillitis alleviation (Fig. 1J). In February 2021, she was admitted to the intensive care unit because of her systemic condition, and her right-eye treatment was discontinued.

## 3 Discussion

**Figure 2:**
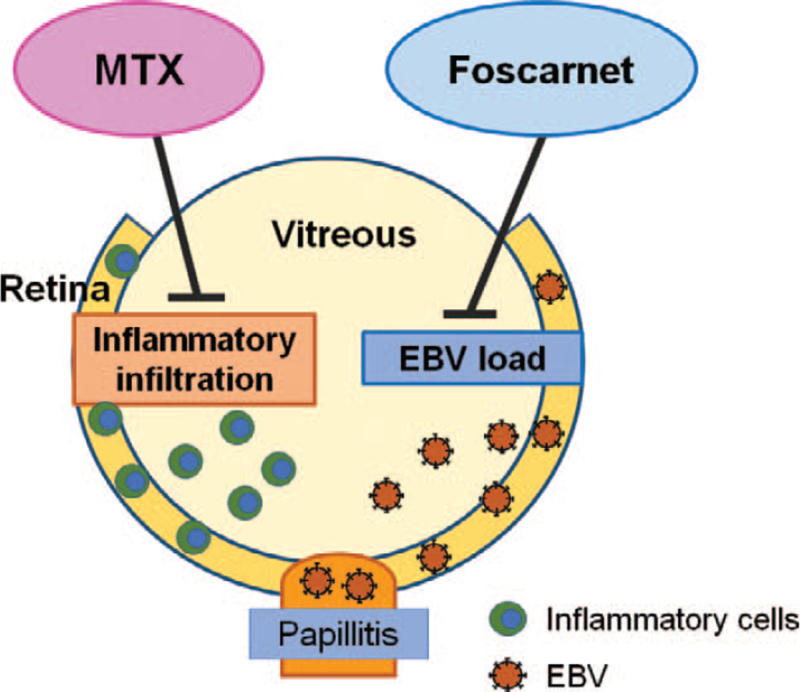
Effects of Foscarnet and Methotrexate (MTX) on Epstein–Barr virus (EBV)-induced intraocular inflammation. Foscarnet reduces the EBV load, but not inflammatory cells. In contrast, MTX reduces the infiltrated inflammatory cells, but not the EBV load. EBV may have originally infected the retina, and in later stages the optic-nerve papillae. EBV may not have infected the infiltrated inflammatory cells into the vitreous and some of the retinal lesions. EBV = Epstein–Barr virus, MTX = methotrexate.

The EBV level reduced after foscarnet injection (Fig. 1K). While vitreous infiltration appeared following foscarnet treatment, it disappeared after injecting MTX. Moreover, intraocular EBV levels elevated under repeated MTX treatments. Thus, foscarnet reduced the EBV load, but not vitreous infiltration (Fig. 2). In contrast, MTX reduced inflammatory vitreous infiltration, but not the EBV load (Fig. 2). The EBV levels increased after the reduction of vitreous infiltration and parallel to papillitis progression and alleviation, suggesting that EBV existed in the optic nerve neurons, but not in the vitreous cells. The latter were most likely composed of lymphocytes that proliferated in response to the viral infection in the retinal and optic neurons. EBV levels were not continuously elevated during the expansion of the retinal lesion as well as at the time of vitreous infiltration. In other words, expanded retinal infiltration not only involved EBV infection to the retinal neurons but also the infiltration of EBV-free inflammatory cells. This finding was consistent with the alleviation of retinal infiltration after MTX injection.

Imai et al. reported that EBV-positive T-cell or NK-cell lymphoproliferative diseases also displayed intraocular inflammation, and were successfully treated by an intraocular injection of MTX.^[1]^ Mashima et al reported that intravitreal MTX possibly decreases EBV-infected B cells, which reduces intravitreal EBV-DNA.^[4]^ The concept of MTX-mediated reduced inflammatory cells was consistent with our observation that reactive inflammatory cells were treated by MTX in the presence of EBV infection. However, MTX did not reduce EBV levels, suggesting that the inflammatory cells in the current study were not infected with EBV, but induced in response to the retinal lesion. MTX may have possibly suppressed EBV-free inflammatory cells. By contrast, foscarnet inhibits EBV DNA polymerase,^[3,7]^ which supports our current observation.

The use of systemic anti-viral and steroid therapies was limited because of the deteriorated systemic condition, leading to unsatisfactory prognosis in the current patient. However, changing viral levels with the alterations in the intravitreal injected drugs demonstrated the differences of the effects and roles of the drugs. Foscarnet may reduce the EB viral levels. In contrast, MTX may suppress the reactive inflammatory cells. Intraocularly infiltrated reactive inflammatory cells may damage the tissue, in addition to the EBV infection to the tissue. Therefore, both drugs were valuable for treating EBV-related intraocular inflammation. Thus, the clinician may utilize both drugs according to the lesions of individual patients. Further observational reports are warranted to reach a consensus on the treatment of intraocular EB viral infection.

## Acknowledgments

The authors appreciate all the medical and co-medical staffs in the Department of Ophthalmology and Operation room of St. Luke's International Hospital.

## Author contributions

Conception: YM, TK, and YO. Data collection: YM. Writing the manuscript: YM, NN and YO. Overall responsibility: YO.

**Investigation:** Yasuaki Mushiga, Tatsunori Komoto.

**Writing – original draft:** Yasuaki Mushiga.

**Writing – review & editing:** Norihiro Nagai, Yoko Ozawa.
